# let-7b-5p suppresses the proliferation and migration of pulmonary artery smooth muscle cells via down-regulating IGF1

**DOI:** 10.1016/j.clinsp.2022.100051

**Published:** 2022-05-27

**Authors:** Yadi Zhang, Sihui Tang, Wanchun Yang, Fangbing Du

**Affiliations:** Department of Respiratory Medicine, The Second People's Hospital of Hefei, Hefei Hospital Affiliated to Anhui Medical University, Hefei, Anhui 230011, China

**Keywords:** Pulmonary artery smooth muscle cells, let-7b-5p, IGF1, Proliferation, Migration

## Abstract

**Objectives:**

Some previous studies indicated that the excessive proliferation and migration of Pulmonary Artery Smooth Muscle Cells (PASMCs) could be observed in pulmonary artery intima after Pulmonary Embolism (PE) occurred. In addition, recent studies identified some miRNAs that are differentially expressed in the blood of PE patients, which might be used as a diagnostic biomarker for PE, including let-7a-5p, let-7b-5p, and miR-150-5p. Hence, the authors sought to explore the effects of let-7b-5p in PASMC proliferation and migration and the corresponding regulatory mechanism.

**Methods:**

Platelet-Derived Growth Factor (PDGF) was utilized to induce the hyper-proliferation model in PASMCs. The mRNA and protein expression levels were detected by RT-qPCR and western blot, respectively. The proliferation of PASMCs was evaluated by the detection of PCNA expression, as well as CCK-8 and Edu assays. Wound healing and Transwell assays were exploited to assess the migration ability of PASMCs. The targets of let-7b-5p were predicted based on two bioinformatics online tools. Dual-luciferase and Ago2 pull-down assays were applied to confirm the interaction between let-7b-5p and IGF1.

**Results:**

40 ng/mL PDGF was selected as the optimal concentration to induce PASMCs. let-7b-5p mimics suppressed the proliferation and migration of PDGF-induced PASMCs, while let-7b-5p inhibitor led to the opposite result. In further mechanism exploration, IGF1 was predicted and confirmed as the direct target gene of let-7b-5p. The promotion role of IGF1 overexpression on the proliferation and migration of PDGF-induced PASMCs was dramatically countered by let-7b-5p mimics.

**Conclusion:**

let-7b-5p prohibits the proliferation and migration of PDGF-induced PASMCs by modulating IGF1.

## Introduction

With the rapid development of sequencing technology, research on the relation between non-coding RNAs (ncRNAs) and human diseases has increased exponentially in the last decades. Micro RNAs (miRNAs), a type of ncRNAs with an approximate length of 22 nt, can suppress the transcription or translation of target mRNAs, thereby regulating human physiological and pathological processes.^1^ To date, the total number of miRNAs identified and validated in the human genome is in excess of 1000.^2^ For example, miR-222 has been proven to be involved in the modulation of insulin sensitivity.^3^ Besides, Jiang, et al.^4^ demonstrated that miR-130a is capable of improving cardiac function in a heart failure model. Nevertheless, there are still significant gaps in comprehensively understanding the roles of miRNAs since human diseases are diverse and complex.

As a potentially lethal complication of venous thromboembolism, Pulmonary Embolism (PE) is the obstruction of pulmonary arteries resulting from thrombus migrating from a deep vein in the legs or pelvis. The incidence of PE in the population is obviously associated with age; from 0.14% of people aged 40‒49 to 1.13% of aged 80 years or over.^5^ With the aging of the global population, the burden of PE is increasing sharply worldwide,^6^ especially in China.^7^ Understanding the pulmonary pathophysiology of PE at an RNA level is significant in developing efficient management and therapies for PE patients.

Some previous studies indicated that the excessive proliferation and migration of Pulmonary Artery Smooth Muscle Cells (PASMCs) could be observed in pulmonary artery intima after PE occurred.^7^^,^^8^ However, there is a little study reporting the mechanism behind these behaviors of PASMCs. In recent years, some miRNAs have been found to be differentially expressed in the blood of PE patients, which might be used as a diagnostic biomarker for PE.^9^^,^^10^ Liu, Liu and Kang^11^ recently identified three miRNAs that were differentially expressed in blood from PE patients, including let-7a-5p, let-7b-5p, and miR-150-5p. let-7b-5p is a well-studied miRNA, which could suppress cell proliferation and migration in various diseases, including gastric cancer^12^ and glioma.^13^ Accordingly, the authors speculated that the abnormal proliferation and migration of PASMCs may be related to the dysregulation of let-7b-5p following PE.

Herein, the authors sought to explore the effect of let-7b-5p on the proliferation and migration of PASMCs and unravel the underlying mechanism.

## Methods

### Cell culture

PASMCs purchased from ATCC (VA, USA) were cultivated in a regular growth medium containing DMEM and 10% FBS in a humidified environment of 5% CO_2_ at 37 °C. After cell confluency reached 80‒90%, cultured cells were digested with 0.25% Trypsin, followed by subculture at a ratio of 1:2. The DMEM, FBS, and 0.25% Trypsin were purchased from Gibco (NY, USA). This study has been approved by the Ethics Committee of the Second People's Hospital of Hefei and Hefei Hospital Affiliated with Auhui Medical University.

### Platelet-derived growth factor (PDGF) treatment

PDGF (Sigma-Aldrich, MO, USA) was utilized to establish an abnormal proliferation model in PASMCs. For PDGF treatment, PASMCs were seeded into 96-well plates and incubated for 24 h, followed by incubation with different concentrations of PDGF (0, 10, 20, and 40 ng/mL). After cultivation for 24h, the proliferation and let-7b-5p expression of cells were measured.

### CCK-8 assay

Treated cells in 96-well plates were gently washed thrice with PBS, and subsequently added with 100 μL CCK-8 reagent diluted in DMEM (1:10, v/v) to further incubate for 2 h. Finally, the absorbance at 450 nm of each well was detected with a Microplate reader (Bio-Rad, CA, USA) to calculate cell viability.

### Reverse transcription‑quantitative polymerase chain reaction (RT-qPCR)

The extraction of total RNA from cells and subsequent reverse transcription were carried out according to standard protocols. RT-qPCR analysis was performed on the 7500 Real-Time PCR System (Applied Biosystems, CA, USA) to measure the expression levels of let-7b-5p, IGF1, GAPDH, and U6. GAPDH and U6 were utilized to normalize the expression of IGF1 and let-7b-5p, respectively; and the expressions were analyzed based on the 2^–ΔΔCt^ method.^14^ The primer sequences used in this study were described as follows: let-7b-5p (Forward 5’- GTGAGGTAGTAGGTTGTGTG -3’; Reverse 5’- GGTCCAGTTTTTTTTTTTTTTTAACCA-3’), IGF1 (Forward 5’- GCTCTTCAGTTCGTGTGTGGA-3’; Reverse 5’- GCCTCCTTAGATCACAGCTCC -3’), GAPDH (Forward 5’- GGAGCGAGATCCCTCCAAAAT- 3’; Reverse 5’- GGCTGTTGTCATACTTCTCATGG-3’).

### Cell transfection

The let-7b-5p mimic, inhibitor, and their corresponding negative control (NC mimic and NC inhibitor), the IGF1 overexpression vector, and blank vector were utilized in this study. Briefly, cells were seeded in plates for 24 h incubation prior to transfection. The transfection in cells was performed with Lipofectamine® 2000 reagent (Invitrogen, CA, USA) mixed with different plasmids. After 48h transfection, transfected cells were harvested to assess the transfection efficiency by RT-qPCR.

### Western blot (WB)

Total protein of cells was extracted using RIPA buffer (Abcam, MA, USA), followed by quantified with a BCA Kit (Beyotime, Shanghai, China). Western blot analysis was conducted as reported by Mahmood and Yang.^15^ Briefly, the proteins were separated by SDS-PAGE gel before being transferred onto a PVDF membrane. Then, the membrane was blocked with skimmed milk for 1.5 h, followed by incubated with primary antibodies overnight and secondary antibodies for 2h. Finally, protein bands were visualized by ECL Substrate Kit (Abcam, MA, USA), and quantified using Image J software. The antibodies used in this study were all purchased from Abcam (MA, USA), of which dilutions were as follows: proliferating cell nuclear antigen (PCNA) (#ab29, 1:2000), GAPDH (#ab8245, 1:8000), IGF1 (#ab134140, 1:20000), and Goat Anti-Rabbit IgG H&L (HRP) (#ab6721, 1:3000).

### Ethynyl deoxyuridine (Edu) assay

EdU Staining Proliferation Kit (Abcam, MA, USA) was utilized to evaluate the proliferation of transfected cells in different groups according to protocols provided by the manufacturer. Briefly, transfected cells were incubated with Edu overnight, then fixed and stained. Cell nuclei were stained with DAPI (Invitrogen, CA, USA) for 15 min prior to observing and counting EdU-positive cells under a BX51 microscope (Olympus, Tokyo, Japan).

### Wound healing assay

A total of 2×10^5^ transfected cells were seeded into each well of six-well plates and cultured until a monolayer of cells had formed. The 200 μL pipette tip was used to create similar size of scratches in the cell layer for each group. Next, scratched cells were removed by gently rinsing PBS thrice; the remaining cells were continually cultured for 24 h. After scratching for 0 and 24 h, the wound of each well was photographed with a BX51 microscope; and the migrated area was calculated to evaluate cell migration ability.

### Transwell migration assay

The migration of transfected cells was also assessed by Transwell chambers (Corning, NY, USA). In brief, 700 μL DMEM with serum was added to the lower chamber. Meanwhile, transfected cells were plated on the upper chambers containing 200 μL DMEM for 24 h incubation. 24 h later, cells still in the upper chamber were wiped out, while cells traversing the membranes to the lower chamber were fixed in 4% paraformaldehyde prior to the staining of 0.1% crystal violet. The stained cells were imaged and counted in five random visual fields under a BX51 microscope.

### Bioinformatics prediction

ENCORI (The Encyclopedia of RNA Interactomes), which is also known as starbase, (http://starbase.sysu.edu.cn)^16^ was utilized to predict the potential target genes of let-7b-5p.

Metascape (https://metascape.org)^17^ was applied to perform the functional annotation of predicted genes. Afterward, the predicted genes associated with “positive regulation of smooth muscle cell proliferation”, “positive regulation of smooth muscle cell migration”, “smooth muscle cell migration”, and “regulation of smooth muscle cell migration” were intersected to obtain objective gene.

### Dual-luciferase assay

To construct the luciferase reporter vector, the 3’UTR (wildtype, WT or mutated control, MUT) of the IGF1 cDNA fragments incorporating the putative let-7b-5p junction site were amplified to insert into the upstream of the reporter gene in the pmirGlo dual-luciferase vector (Addgene, MA, USA). For luciferase assay, 293T cells cultured in a 24-well plate were co-transfected with the constructed vectors (IGF1 3’UTR WT/MUT) containing firefly luciferase and let-7b-5p mimics or the negative control (NC mimics). After transfection, the Firefly fluorescence was normalized to the Renilla fluorescence to calculate the luciferase activity.

### Ago2 pull down

By using a Magna RIP Kit (Millipore, MA, USA), the Ago2 pull-down assay was carried out following the guidelines provided by manufacturers. Cells were lysed, and then immunoprecipitated with magnetic beads conjugated with antibodies against Ago2 (#ab32381, Abcam, MA, USA). Immunoprecipitated RNA was analyzed qRT-PCR. IgG was served as a negative control.

### Statistical analysis

All statistical analysis was carried out on GraphPad Prism 8.0.1. In this study, all experiments were repeated at least thrice, and all data were presented as mean ± Standard Deviation (SD). A students’ test or one-way ANOVA with post-hoc test (Bonferroni) was performed to analyze the discrepancy among the groups. It is considered to be statistically discrepant when *p* < 0.05.

## Results

### PDGF induces PASMCs abnormal proliferation with concomitantly decreasing let-7b-5p expression in PASMCs

**Fig. 1:**
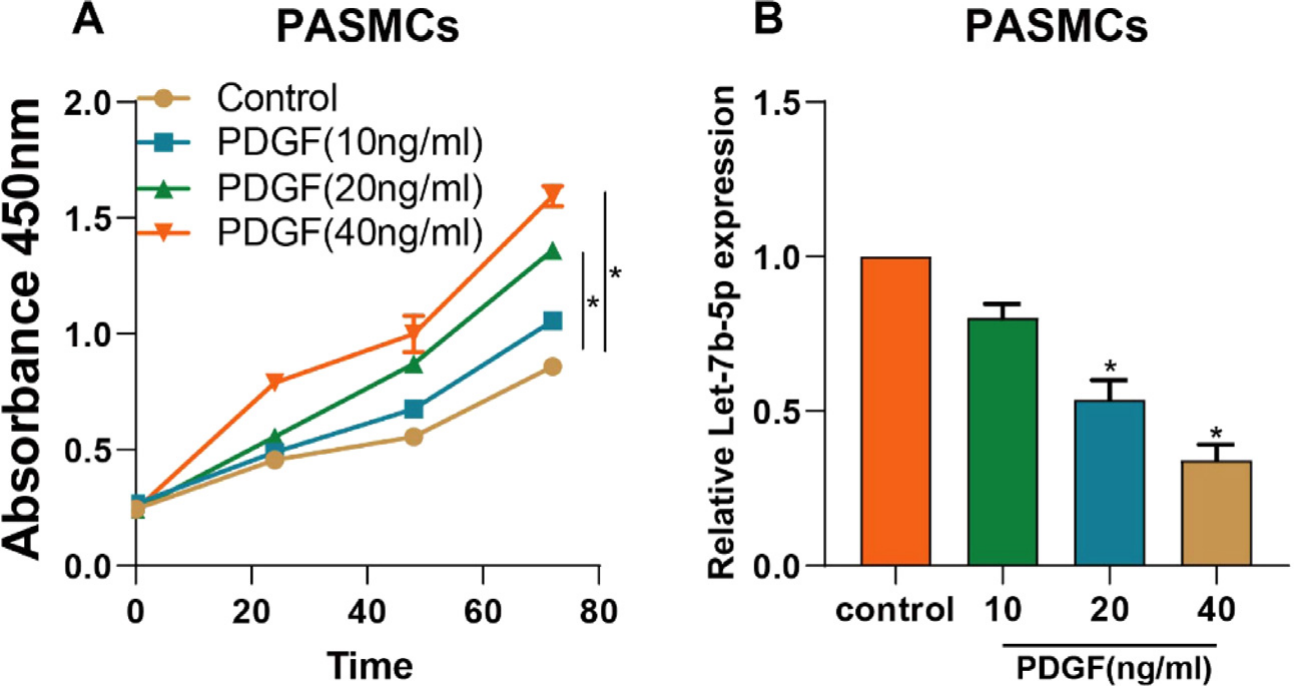
PDGF induced the abnormal proliferation of PASMCs and the downregulation of let-7b-5p. (A) The proliferation of PASMCs with different concentrations of PDGF detected by CCK-8 assay. (B) The expression of let-7b-5p in PASMCs with different concentrations of PDGF determined by RT-qPCR. Note: *p < 0.05.

To study the relation of let-7b-5p expression with cell proliferation in PASMCs, the expression level of let-7b-5p was detected following treatment with different doses of PDGF in PASMCs. Compared with the control group (0 ng/mL PDGF), the proliferation of PASMCs was promoted by PDGF in a dose-dependent way (Fig. 1A). However, the expression level of let-7b-5p expression was decreased under the administration of PDGF (Fig. 1B). Among diverse concentrations, 40 ng/mL of PDGF exhibited the strongest suppression effect on let-7b-5p expression. Hence, the authors chose the 40 ng/mL as the optimal concentration for the subsequent experiments.

### PDGF-induced abnormal proliferation and migration of PASMCs were antagonized by let-7b-5p mimics, reinforced by let-7b-5p inhibitor

**Fig. 2:**
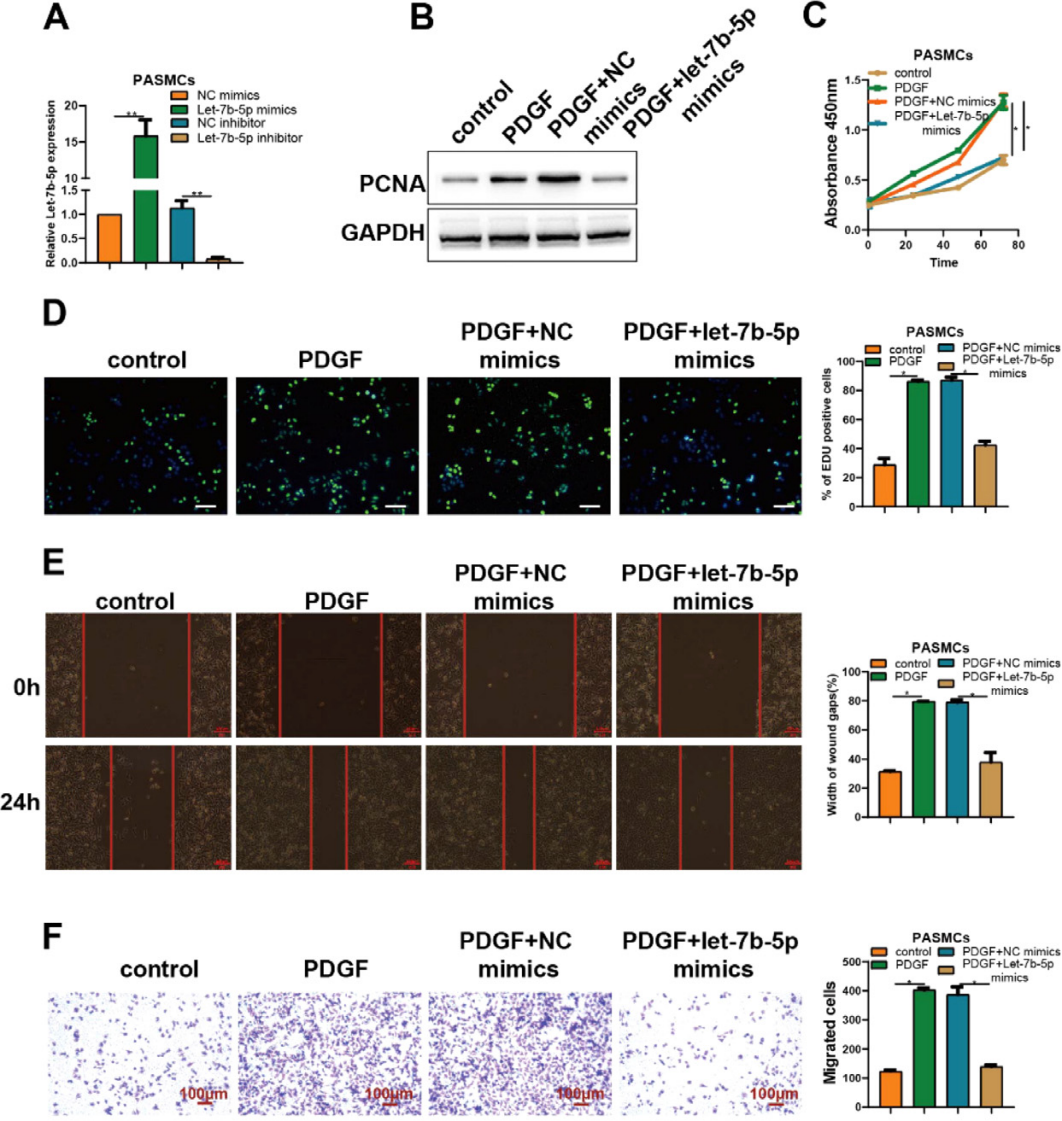
The overexpression of let-7b-5p could antagonize PDGF-induced abnormal proliferation and migration of PASMCs. (A) The transfection efficiency of let-7b-5p mimics and inhibitor verified by RT-qPCR. (B) The PCNA protein expression in PASMCs with different treatments detected by western blot. The proliferation of PASMCs with different treatments detected by (C) CCK-8 and (D) Edu assays (scale bar = 100μm). (E) Wound healing and (F) Transwell assays detected the migration ability of PASMCs with different treatments; Scale bar = 100μm. Note: *p < 0.05.

To better understand the role of let-7b-5p on abnormal proliferation and migration of PASMCs, let-7b-5p mimics or let-7b-5p inhibitor was utilized to transfect into PASMCs. RT-qPCR analysis verified the successful transfection of let-7b-5p mimics and let-7b-5p inhibitor in PASMCs (Fig. 2A).

Compared with the control group, the expression levels of PCNA in PASMCs were obviously elevated by the treatment of PDGF. Notably, the increased expression levels of PCNA induced by PDGF were blocked by the transfection of let-7b-5p mimics (Fig. 2B). Consistent with previous results, PDGF treatment significantly increased the proliferation of PASMCs which was almost reversed by the transfection of let-7b-5p mimics, as confirmed by CCK-8 and Edu assays (Fig. 2C and D). Moreover, both wound healing and Transwell assays showed that the treatment of PDGF remarkably induced the migration of PASMCs which could be blocked by the transfection of let-7b-5p mimics (Fig. 2E and F). These results suggested the suppression role of let-7b-5p on the abnormal proliferation and migration of PASMCs induced by PDGF.

**Fig. 3:**
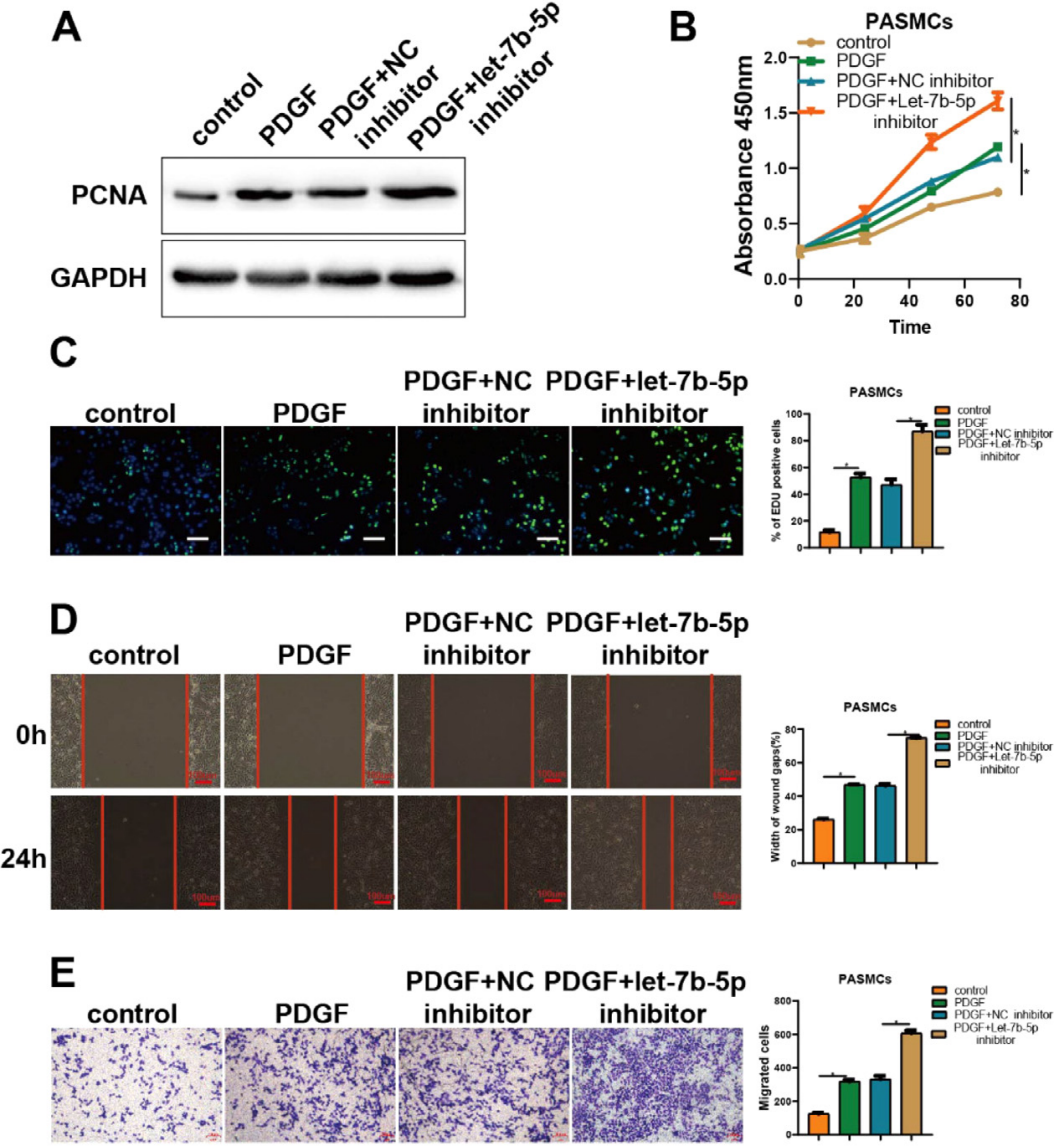
Suppressing let-7b-5p could reinforce PDGF-induced abnormal proliferation and migration of PASMCs. (A) The PCNA protein expression in PASMCs with different treatments detected by western blot. The proliferation of PASMCs with different treatments detected by (B) CCK-8 and (C) Edu assays (scale bar = 100μm). (D) Wound healing and (E) Transwell assay detected the migration ability of PASMCs with different treatments; Scale bar = 100μm. Note: *p < 0.05.

The results of experiments regarding let-7b-5p inhibitor corroborated the above findings (Fig. 3). As shown by PCNA detection, CCK-8, and Edu assays, the promotion of PDGF on cell proliferation in PASMCs was significantly strengthened by the transfection of let-7b-5p inhibitor (Fig. 3A-C). Besides, wound healing and Transwell assays indicated that the transfection of let-7b-5p inhibitor also markedly potentiated the promotion of PDGF on migration in PASMCs (Fig. 3D and E). Hence, let-7b-5p could reverse the aberrant proliferation and migration of PASMCs induced by PDGF.

### let-7b-5p directly interacts with IGF1, and down-regulates the expression of IGF1

**Fig. 4:**
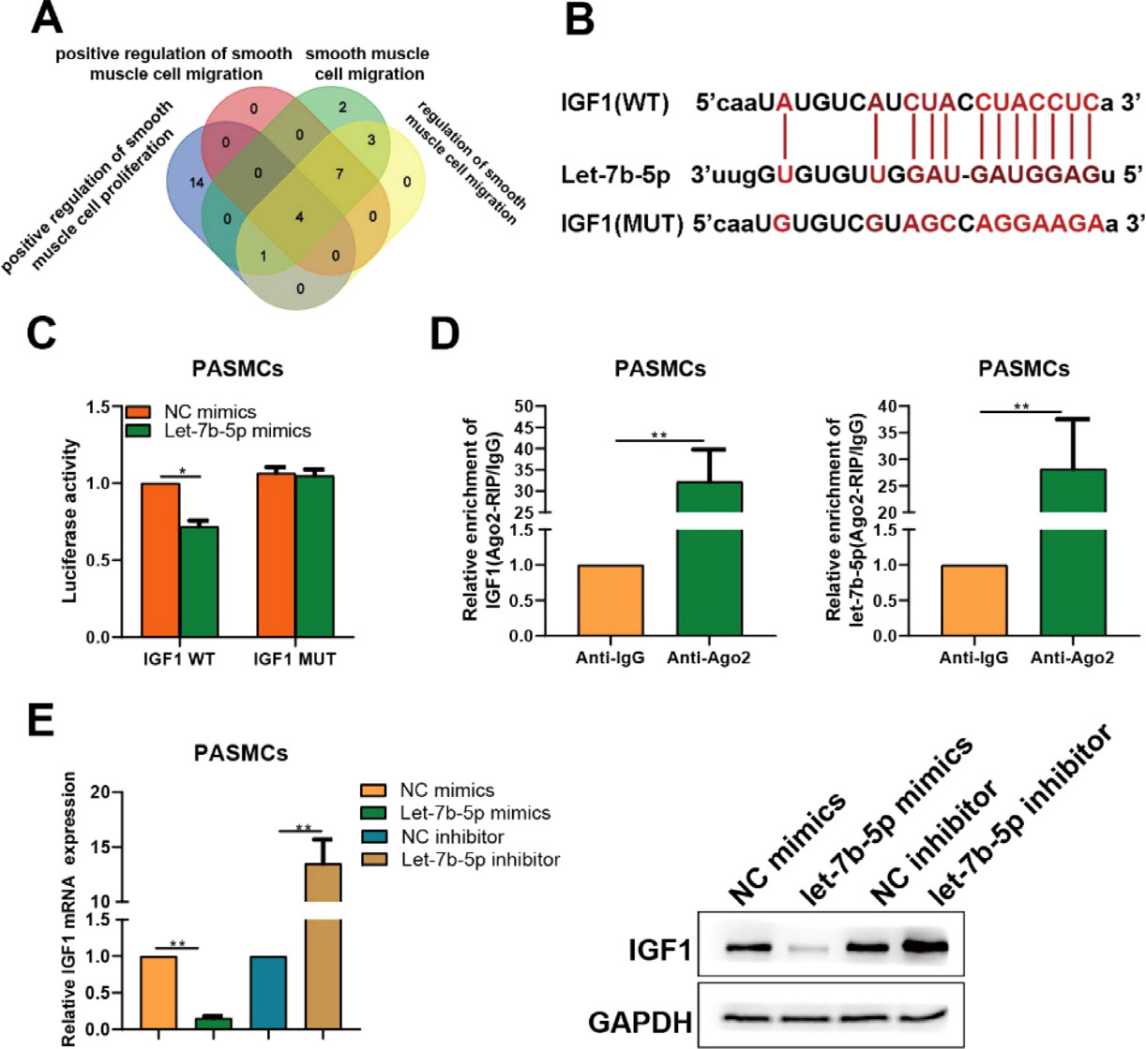
IGF1 is a direct target of let-7b-5p in PASMCs. (A) Based on the prediction result of starbase, four target genes of let-7b-5p further identified by intersecting functional annotation results. (B) Potential binding sites between IGF1 and let-7b-5p. (C) Dual-luciferase and (D) Ago2 pull down assays indicated the direct interaction between IGF1 and let-7b-5p. (E) The mRNA (left) and protein (right) expression levels of IGF1in PASMCs after transfection with let-7b-5p mimics or inhibitor. Note: *p < 0.05 and ^⁎⁎^*p* < 0.01.

To uncover the mechanism behind the effects of let-7b-5p on PASMCs, the target genes of let-7b-5p were identified by bioinformatics tools. A total of 31 potential target genes predicted by starbase were uploaded to Metascape to conduct functional annotation. Then, the genes overlapped in the functional terms of “positive regulation of smooth muscle cell proliferation”, “positive regulation of smooth muscle cell migration”, “smooth muscle cell migration”, and “regulation of smooth muscle cell migration” were obtained, including IGF1, PAK1, PDGFB, and ADAMTS1 (Fig. 4A). Given that IGF1 has been reported to be involved in the regulation of PASMCs proliferation.^18^ Hence, IGF1 was chosen as the studied object for further analysis. Dual-luciferase assay was performed to confirm the relation between let-7b-5p and IGF1. The potential binding sites between let-7b-5p and IGF1 were depicted in Fig. 4B. Dual-luciferase assay verified that let-7b-5p reduced the luciferase activity of the IGF1 wild-type group, but not the IGF1 mutant group (Fig. 4C). Ago2 pull-down assay further confirmed that IGF1 could spatially interact with let-7b-5p via Ago2 protein (Fig. 4D).

Afterward, the authors also investigated the effect of let-7b-5p on IGF1expression in PASMCs. Compared with the control group, the transfection of let-7b-5p mimics observably reduced the expression of IGF1 in PASMCs, while the let-7b-5p inhibitor exerted the converse effect (Fig. 4E). These results revealed that let-7b-5p directly interacts with IGF1, and further down-regulating the expression of IGF1 in PASMCs.

### IGF1 functions as a target of let-7b-5p on the proliferation and migration of PASMCs

**Fig. 5:**
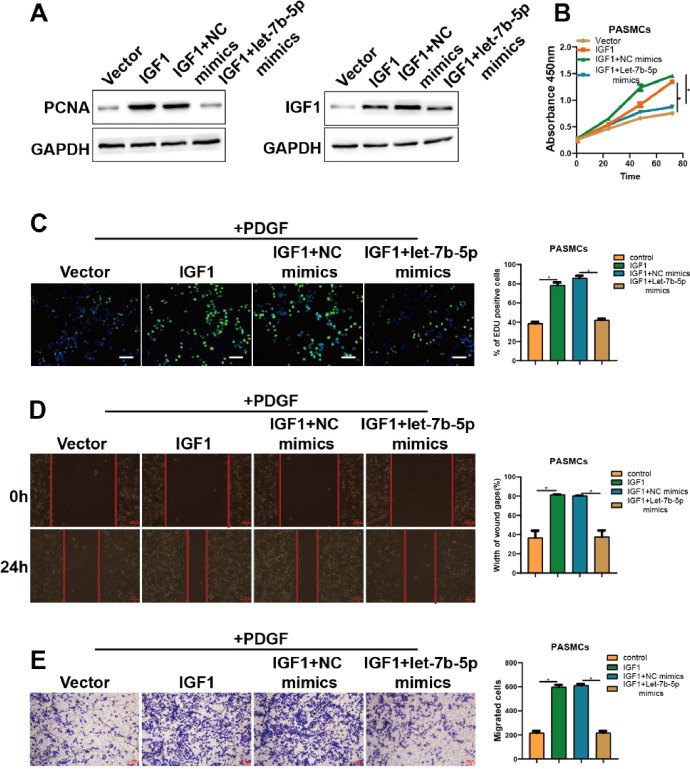
let-7b-5p reverses the abnormal proliferation and migration promoted by the IGF1. (A) The protein expression of PCNA and IGF1 in PDGF-induced PASMCs with different transfections detected by western blot. The proliferation of PDGF-induced PASMCs with different treatments detected by (B) CCK-8 and (C) Edu assays (scale bar = 100μm). (D) Wound healing and (E) Transwell assay detected the migration ability of PDGF-induced PASMCs with different treatments; Scale bar = 100μm. Note: *p < 0.05.

To further investigate whether the inhibitory effect of let-7b-5p on PDGF-induced proliferation and migration of PASMCs was mediated by IGF1, the IGF1 overexpression vector was transfected into PASMCs alone or combined with let-7b-5p before treating PASMCs with PDGF. As shown in Fig. 5A, IGF1 overexpression significantly increased PCNA expression in PDGF-induced PASMCs, whereas this effect was greatly attenuated by the introduction of let-7b-5p mimics (Fig. 5A). CCK-8 and Edu results showed that overexpressing IGF1 significantly promoted the proliferation of PDGF-induced PASMCs, which was countered after co-transfected with let-7b-5p mimics (Fig. 5B and C). The overexpression of IGF1 also strengthened the migration ability of PDGF-induced PASMCs (Fig. 5D and E). However, the effect of IGF1 overexpression on PDGF-induced PASMCs migration was substantially suppressed by let-7b-5p mimics (Fig. 5D and E), suggesting that let-7b-5p is involved in the migration of PDGF-induced PASMCs mediated by IGF1. These results revealed that let-7b-5p up-regulation blocked IGF1-mediated promotion of proliferation and migration in PDGF-induced PASMCs.

## Discussion

A growing number of research revealed that the excessive cellular proliferation and enhancing migration ability of PASMCs are associated with the pulmonary vasoconstriction and remodeling following PE.^19^^,^^20^. Here, the authors reported that let-7b-5p is able to suppress the excessive proliferation and migration of PDGF-induced PASMCs via targeting IGF1.

PDGF is known as a polypeptide growth factor that regulates cell growth and division, and has been identified as a major mitogen for smooth muscle cells.^21^^,^^22^ It has been reported that the up-regulation of PDGF is closely related to the progression of PE, such as pulmonary hypertension.^23^ In this study, PDGF was utilized to stimulate PASMCs *in vitro*. Consistent with previous studies,^24^ the present data showed that PDGF significantly promoted proliferation in a dose-dependent way. Notably, the expression levels of let-7b-5p in PASMCs decreased with the increase of PDGF concentration, which suggested that let-7b-5p may be involved in the abnormal proliferation of PASMCs. Research on the pathology of multiple cancers has indicated that let-7b-5p plays an inhibitory role in tumorigenesis. Xu, et al.^25^ revealed that let-7b-5p could significantly suppress the malignant phenotype of multiple myeloma cells. Huang, et al.^26^ indicated that let-7b-5p could repress cell proliferation and invasion, as well as the tumor formation of castration-resistant prostate cancer. Accordingly, the authors suppose that let-7b-5p participates in the abnormal proliferation and migration of PASMCs. In line with our assumption, both CCK-8 and Edu assays revealed that the excessive proliferation of PDGF-induced PASMCs was potently countered by overexpressing let-7b-5p, while reinforced by silencing let-7b-5p. As known, PCNA is a standard marker commonly utilized to assess cell proliferation since its critical role in DNA replication.^27^ The authors subsequently detected the PCNA expression levels in PASMCs from each group, with results that validated the finding above. Moreover, wound healing and Transwell migration assays demonstrated the overexpression of let-7b-5p reversed the promoting migration effect of PDGF in PASMCs. Conversely, the deletion of let-7b-5p aggravated the effect of PDGF on the migration of PASMCs. Combined with the above results, the present study indicated that let-7b-5p is capable of impeding PDGF-induced abnormal proliferation and migration in PASMCs.

It has been widely reported that miRNAs have major effects on multiple biological processes, including cell proliferation, development, and apoptosis, by regulating target genes at the post-transcriptional level.^28^ Thus, the authors subsequently predicted the potential target of let-7b-5p using bioinformatics online tools to understand the mechanism behind its inhibitory role in the proliferation and migration of PDGF-induced PASMCs. Then, a total of four genes were identified as potential target genes of let-7b-5p that are involved in the biological process of PASMCs, which included IGF1, PAK1, PDGFB, and ADAMTS1. IGF1 is a known mitogen that can be found in most cell types, including smooth muscle cells. Several studies reported that IGF1 plays a crucial role in regulating the proliferation of vascular smooth muscle cells.^29^^,^^30^ Besides, a previous study demonstrated that IGF1 played an anti-apoptosis effect on PASMCs, thereby promoting the accumulation of PASMCs during pulmonary arterial hypertension.^31^ Based on these, the authors finally selected IGF1 as the studied target of let-7b-5p for further analysis. As expected, the present study confirmed that let-7b-5p directly interacted with the 3’-UTR of IGF1 to suppress the expression of IGF1 in PASMCs. In the present study, the overexpression of IGF1 promoted the proliferation and motility of PASMCs, consistent with the known function of IGF1 in vascular smooth muscle cells. Additionally, such effects of IGF1 overexpression on the proliferation and migration of PASMCs were observably restrained when co-transfected with let-7b-5p mimics, further indicating that the suppression role of let-7b-5p on the excessive proliferation and migration of PASMCs was exerted by targeting IGF1. Collectively, the present study facilitates an understanding of the effects of let-7b-5p on PASMCs and the mechanism by which let-7b-5p against the aberrant proliferation and migration of PASMCs induced by PDGF. However, it should be acknowledged that the major limitation of this study is lacking validation of the effect of let-7b-5p in the progression of PE *in vivo*.

In summary, the present study first reveals the suppression role of let-7b-5p on the abnormal proliferation and migration of PASMCs induced by PDGF and found that IGF1 serves as the downstream effector in this process. The authors hope that the present findings could improve the understanding of pathogenesis and the development of novel therapeutic targets in PE.

## Authors' contributions

Yadi Zhang and Fangbing Du conceived the project, and designed the experiments. Yadi Zhang, Sihui Tang, Wanchun Yang, and Fangbing Du performed the experiments, and analyzed the data. Yadi Zhang and Fangbing Du wrote and revised the manuscript.

## Funding

This research did not receive any specific grant from funding agencies in the public, commercial, or not-for-profit sectors.

## Conflicts of interest

The authors declare no conflicts of interest.
